# 贝伐单抗联合化疗治疗非小细胞肺癌的*meta*分析

**DOI:** 10.3779/j.issn.1009-3419.2013.02.05

**Published:** 2013-02-20

**Authors:** 涛 张, 帅飞 袁, 子平 王, 茜 张, 盼盼 赵, 莉 单

**Affiliations:** 1 830011 乌鲁木齐，新疆医科大学附属肿瘤医院内一科 Department of Medical Oncology, Tumor Hospital Affiliated to Xinjiang Medical University, Urumqi 830011, China; 2 100021 北京，中国医学科学院北京协和医学院肿瘤医院内科 Department of Medical Oncology, Cancer Hospital & Institute, Academy of Medical Sciences and Peking Union Medical College, Beijing 100021, China; 3 830011 乌鲁木齐，新疆医科大学附属肿瘤医院研究生管理科 Department of Graduate, Tumor Hospital Affiliated to Xinjiang Medical University, Urumqi 830011, China

**Keywords:** 贝伐单抗, 化疗, 肺肿瘤, *Meta*分析, Bevacizumab, Chemotherapy, Lung neoplasms, *Meta* analysis

## Abstract

**背景与目的:**

贝伐单抗是抑制血管内皮生长因子受体（vascular endothelial growth factor receptor, VEGFR）的重组人源化单克隆抗体，本研究旨在系统评价贝伐单抗联合化疗治疗非小细胞肺癌（non-small cell lung cancer, NSCLC）疗效及安全性。

**方法:**

计算机检索中国生物医学文献数据库（CBM）、中国期刊全文数据库（CNKI）、维普数据库（VIP）、万方数据库、The Cochrane Library、PubMed、Ovid、EMBASE及SCI等数据库，收集有关贝伐单抗联合化疗治疗NSCLC的随机对照试验（randomized control trial, RCT）；主要结局指标包括有效率、无进展生存时间（progression free survival, PFS）、总生存期（overall survival, OS）、治疗相关死亡率及毒性反应；采用相对危险度（relative risk, RR）和风险比（hazard ratios, HR）为效应量，各效应量以95%置信区间（95%CI）表示，Stata 12.0统计软件进行*meta*分析。

**结果:**

共纳入6项RCT，共2, 338例晚期NSCLC患者，*meta*分析结果显示，与单纯化疗方案比较，贝伐单抗（7.5 mg/kg或15 mg/kg）联合化疗方案可提高晚期NSCLC的有效率（RR=1.68, *P* < 0.01, 95%CI: 1.31-2.15; RR=1.79, *P* < 0.01, 95%CI: 1.53-2.08），降低疾病进展风险（HR=0.75, *P* < 0.01, 95%CI: 0.61-0.89; HR=0.69, *P* < 0.01, 95%CI: 0.62-0.77）和疾病死亡风险（HR=0.94, *P* < 0.01, 95%CI: 0.77-1.10; HR=0.87, *P* < 0.01, 95%CI: 0.78-0.97）；高剂量贝伐单抗（15 mg/kg）联合化疗方案增加了晚期NSCLC患者的治疗相关死亡率（RR=1.88, *P*=0.01, 95%CI: 1.16- 3.05）及其它毒性反应的发生率。

**结论:**

无论一线还是二线治疗，贝伐单抗联合化疗方案可提高晚期NSCLC患者的有效率、PFS及OS。

## Abstract (en)

**Background and objective:**

Bevacizumab is a recombinant, humanised, monoclonal antibody against the vascular endothelial growth factor receptor (VEGFR), the aim of this *meta*-analysis is to evaluate the clinical efficacy and safety of bevacizumab combined with chemotherapy for non-small cell lung cancer (NSCLC).

**Methods:**

The CBM, CNKI, VIP, WanFang, The Cochrane Library, PubMed, Ovid, EMBASE and SCI *etc* were retrieved by computer, the randomized controlled trials (randomized control trial, RCT) of bevacizumab combined with chemotherapy in the treatment of NSCLC were collected by us. The outcomes included overall survival (OS), progression-free survival (PFS), response rate (RR), toxicities and treatment related mortality. Relative risk (RR) and hazard ratios (HR) were used for the *meta*-analysis and were expressed with 95% confidence intervals (95%CI), and the software Stata 12.0 was used for *meta*-analyses.

**Results:**

Six trials and 2, 338 advanced NSCLC patients were included. *Meta* analysis indicated that compared with chemotherapy alone, the bevacizumab (7.5 mg/kg or 15 mg/kg) combination with chemotherapy could increase overall survival rate (RR=1.68, *P* < 0.01, 95%CI: 1.31-2.15; RR= 1.79, *P* < 0.01, 95%CI: 1.53-2.08), and could decrease the progression of the disease risk (HR=0.75, *P* < 0.01, 95%CI: 0.61-0.89; HR=0.69, *P* < 0.01, 95%CI: 0.62-0.77) and the risk of disease death (HR=0.94, *P* < 0.01, 95%CI: 0.77-1.10; HR=0.87, *P* < 0.01, 95%CI: 0.78-0.97). The high dose of bevacizumab (15 mg/kg) combination with chemotherapy could increase the treatment related mortality (RR=1.88, *P*=0.010, 95%CI: 1.16-3.05) and the rates of other toxic reaction.

**Conclusion:**

Whether first-line or second-line treatment, the addition of bevacizumab to chemotherapy in patients with advanced NSCLC prolongs RR, OS and PFS.

目前肺癌是全球范围内死亡率最高的恶性肿瘤之一，约有80%为非小细胞肺癌（non-small cell lung cancer, NSCLC），其中65%以上的患者在诊断出该病时已为Ⅲb期-Ⅳ期，丧失手术治疗时机。化疗为主的综合抗肿瘤模式是晚期NSCLC的治疗手段，铂类药物联合第三代细胞毒药物是化疗基础，但治疗效果却不尽如人意。因此想要提高晚期NSCLC患者的生存率，需要尝试与现有治疗机制不同的新药物和化疗方案。贝伐单抗是抑制血管内皮生长因子受体（vascular endothelial growth factor receptor, VEGFR）的单克隆抗体，美国批准的贝伐单抗联合紫杉醇及卡铂方案，欧洲批准的贝伐单抗联合含铂两药方案可作为晚期NSCLC患者的一线治疗选择^[1]^。本研究通过检索中英文数据库收集资料，应用*meta*分析的方法对有关贝伐单抗联合化疗的临床随机对照试验（randomized control trial, RCT）进行综合分析，旨在为贝伐单抗联合化疗治疗晚期NSCLC提供更多循证医学依据。

## 1 资料与方法

### 1.1 纳入标准

#### 1.1.1 研究类型

公开发表的RCT，无论是否采用盲法和分配隐藏，语种限定为中文和英文，发表日期截止于2012年10月1日。

#### 1.1.2 研究对象

① 经病理或细胞学确诊的NSCLC患者；②TNM分期在Ⅲb期及以上；③ECOG评分0分-2分；④血常规、肝肾功能及心电图等均未见明显异常。

#### 1.1.3 干预措施

对照组患者接受以铂类为基础的两药联合方案；观察组患者在此基础上联合贝伐单抗治疗，不限制贝伐单抗使用剂量。

#### 1.1.4 结局指标

有效率、无进展生存时间（progression free survival, PFS）、总生存时间（overall survival, OS）、治疗相关死亡率及毒性反应。

### 1.2 排除标准

① 非RCT研究；②综述性文献；③重复发表的文献；④同时接受放疗或者其他治疗方案的研究；⑤当多篇涉及同一研究时，以最近发表的文献为准。

### 1.3 检索策略

计算机检索相关数据库，中文数据库包括中国生物医学文献数据库（CBM）、中国期刊全文数据库（CNKI）、维普数据库（VIP）及万方数据库；英文数据库包括The Cochrane Library、PubMed、Ovid、EMBASE及SCI。检索采用主题词与自由词相结合的方式，检索词主要包括：肺癌、非小细胞肺癌、化疗、贝伐单抗、阿瓦斯汀、安维汀和lung cancer、non-small cell lung cancer、NSCLC、chemotherapy、Bevacizumab、Avastin；对纳入文献的参考文献进一步扩大检索。

### 1.4 文献筛选

按照预先设计的信息提取表，由两名研究者独立检索文献，然后对题目及摘要符合纳入/排除标准的文献进行评价，若题目及摘要难以判断的则查阅全文进行核实，意见不一致时由第三方决定是否采用。

### 1.5 资料提取

资料提取亦由两名研究者独立完成，如遇分歧则由第三方最终决定。提取信息主要包括：①题目、作者、发表日期及来源等；②患者的性别、年龄、病理类型、TNM分期及具体干预措施等；③结局指标；④方法学质量评价的指标。

### 1.6 质量评价

按Cochrane协作网偏倚风险评价标准^[2]^对纳入的临床试验进行方法学质量评价。由两名研究者采用统一的方法独立地对符合纳入标准的文献进行质量学评价，包括随机方法、盲法、分配隐藏、有无失访或退出及意向性分析（intentional analysis, ITT analysis）等5方面，当意见不一致时由第三方决定。上述标准均为“正确或充分”者，发生各种偏倚的可能性最小，其质量为“A”级；有1项或1项以上标准未描述则为不清楚，部分满足，有发生相应偏倚的中度可能性，质量为“B”级；有1项或1项以上标准不正确或未使用，有发生相应偏倚的高度可能性，质量为“C”级。

### 1.7 统计学方法

对收集的数据采用Stata 12.0软件进行*meta*分析。采用相对危险度（relative risk, RR）和风险比（hazard ratios, HR）为效应量，PFS、OS等生存指标由疾病进展风险（HR for disease progression）、疾病死亡风险（HR for disease death）进行合并分析，各效应量以95%置信区间（95%CI）表示。采用卡方检验分析各资料结果的异质性，同时采用*I*^2^对异质性进行定量分析，*I*^2^＞50%时提示研究结果间存在异质性。异质性检验结果无统计学意义采用固定效应模型进行*meta*分析；存在异质性时首先分析产生异质性的原因，如果存在临床异质性则进行亚组分析；如果经过处理后异质性仍无法消除，则采用随机效应模型进行合并分析；如果两组间异质性过大或存在明显临床异质性时，采用描述性分析；*P*＜0.05为差异有统计学意义。

## 2 结果

### 2.1 检索结果

本研究共检出相关文献733篇，包括中文191篇，英文542篇，利用医学文献王软件去重538篇；通过阅读文献题目和摘要，初步获得中文献7篇，英文文献21篇；再次通读全文，排除非RCT及相同研究的文献22篇，最终纳入6项研究^[3-8]^，共2, 338例晚期NSCLC患者（图 1）。

**1:**
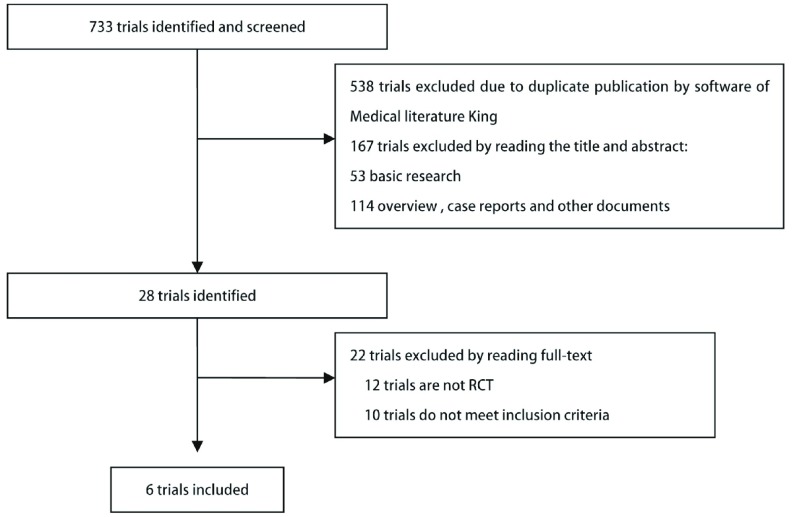
文献检索及筛选流程图 Flow of identification and inclusion of trials

### 2.2 纳入研究的基本特征

纳入的6项RCT中包括4项Ⅱ期临床研究^[3, 5, 7, 8]^和2项Ⅲ期临床研究^[4, 6]^，其中1项临床研究^[5]^在二线治疗时采用贝伐单抗联合化疗，另5项研究^[3, 4, 6-8]^均为一线使用贝伐单抗联合化疗，但Sandler等研究^[4]^中有97例、Reck等研究^[6]^中有60例、Niho等研究^[8]^中有15例术后复发患者接受一线治疗（表 1）。

**1:** 纳入研究的基本特征 The basic characteristics of included trials

Trials	Arm	Case	Gender (M/F)	Histology	Stage （Ⅲb/Ⅳ）	Treatment
Johnson 2004^[3]^		99	63/36	adenocarcinoma, large cell carcinoma, squamous cell carcinoma, other	15/84	
	Control	32	24/8		6/26	TP
	Observation 1	32	20/12		2/30	TP+B (7.5 mg/kg)
	Observation 2	35	19/16		7/28	TP+B (15 mg/kg)
Sandler 2006^[4]^		850	463/387	adenocarcinoma, large cell carcinoma, bronchioloalveolar carcinoma, other	105/647	
	Control	433	253/180		55/337	TP
	Observation	417	210/207		50/310	TP+B (15 mg/kg)
Herbst 2007^[5]^		81	48/33	large cell carcinoma, adenocarcinoma, other	—	
	Control	41	25/16		—	D or P+Placebo
	Observation	40	23/17		—	D or P+B (15 mg/kg)
Reck 2010^[6]^		1, 043	665/378	adenocarcinoma, large cell carcinoma, other	159/802	
	Control	347	223/124		55/266	GP+Placebo
	Observation 1	345	223/122		48/267	GP+B (7.5 mg/kg)
	Observation 2	351	219/132		56/269	GP+B (15 mg/kg)
Soria 2011^[7]^		85	47/38	adenocarcinoma, bronchioloalveolar carcinoma, large cell carcinoma, other	3/82	
	Control	41	24/17		1/40	TP
	Observation	44	23/21		2/42	TP+B (15 mg/kg)
Niho 2012^[8]^		180	115/65	adenocarcinoma, large cellcarcinoma, other	40/125	
	Control	59	38/21		12/42	TP
	Observation	121	77/44		28/83	TP+B (15 mg/kg)
M: male; F: female; TP: Paclitaxel (200 mg/m^2^) combine with Carboplatin (AUC=6); D: Docetaxel (75 mg/m^2^); P: Pemetrexed (500 mg/m^2^); GP: Gemcitabine (1, 250 mg/m^2^) combine with Cisplatin (80 mg/m^2^); B: Bevacizumab.						

### 2.3 纳入研究的质量评价

见表 2。纳入的6项RCT中，1项研究^[6]^被评为“A”级，3项研究^[4, 5, 8]^被评为“B”级，其余皆被评为“C”级；Reck等的研究^[6]^合理地实施了分配隐藏及盲法，其余研究均无明确描述分配隐藏和盲法；综合评价*meta*分析可信度尚可。

**2:** 纳入研究的质量评价 Quality evaluation of included trials

Trials	Randomization	Allocation concealment	Blindness	Follow up	ITT analysis	Baseline	Quality grading
Johnson 2004^[3]^	Computerized	Unclear	No	Yes	Yes	Yes	C
Sandler 2006^[4]^	Stratified	Unclear	Unclear	Yes	Yes	Yes	B
Herbst 2007^[5]^	Stratified	Unclear	Unclear	Yes	Yes	Yes	B
Reck 2010^[6]^	Stratified	Yes	Double-blind	Yes	Yes	Yes	A
Soria 2011^[7]^	Unclear	Unclear	No	Yes	Yes	Yes	C
Niho 2012^[8]^	Stratified	Unclear	Unclear	Yes	Yes	Yes	B

### 2.4 异质性检验

分别以有效率、PFS、OS、治疗相关死亡率及毒性反应发生率进行效应指标的异质性检验，低剂量组高血压的发生率存在异质性（*P*＜0.05），采用随机效应模型进行*meta*分析；其余结局指标无异质性（*P*＞0.05），采用固定效应模型进行*meta*分析。

### 2.5 *meta*分析结果

#### 2.5.1 有效率

6项研究^[3-8]^均报道了有效率，*meta*分析结果（图 2）显示，低剂量贝伐单抗（7.5 mg/kg）或高剂量贝伐单抗（15 mg/kg）联合化疗方案治疗晚期NSCLC的有效率，与单纯使用化疗方案相比，差异具有统计学意义（RR=1.68, *P*＜0.01, 95%CI: 1.31-2.15; RR=1.79, *P*＜0.01, 95%CI: 1.53-2.08）。因Herbst等的研究^[5]^为二线方案，故剔除该研究以后，贝伐单抗联合化疗方案一线治疗晚期NSCLC的有效率，与单纯使用化疗方案比较，差异具有统计学意义（RR=1.78, *P*＜0.01, 95%CI: 1.56-2.03）。

**2:**
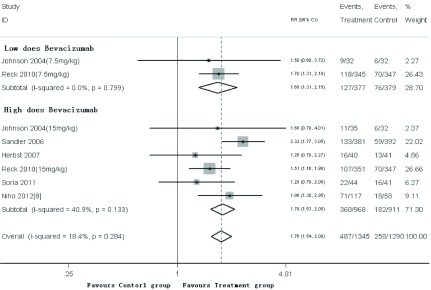
有效率的*meta*分析 *Meta*-analysis of Efficient

#### 2.5.2 PFS

6项研究^[3-8]^均报道了PFS，*meta*分析结果（图 3）显示，低剂量贝伐单抗（7.5 mg/kg）或高剂量贝伐单抗（15 mg/kg）联合化疗方案可降低晚期NSCLC患者的疾病进展风险，差异具有统计学意义（HR=0.75, *P*＜0.01, 95%CI: 0.61-0.89; HR=0.69, *P*＜0.01, 95%CI: 0.62-0.77）。剔除Herbst等的研究^[5]^后，贝伐单抗联合化疗方案一线治疗晚期NSCLC的PFS，与单纯使用化疗方案比较，差异具有统计学意义（RR=0.71, *P*＜0.01, 95%CI: 0.64-0.78）。

**3:**
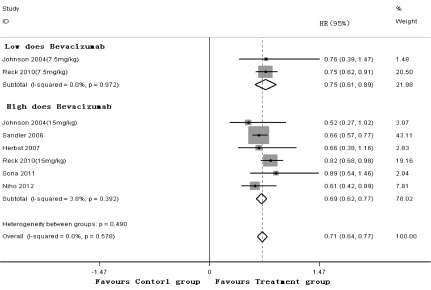
PFS的*meta*分析 *Meta*-analysis of PFS

#### 2.5.3 OS

6项研究^[3-8]^均报道了OS，*meta*分析结果（图 4）显示，低剂量贝伐单抗（7.5 mg/kg）或高剂量贝伐单抗（15 mg/kg）联合化疗方案可降低晚期NSCLC患者的疾病死亡风险，差异具有统计学意义（HR=0.94, *P*＜0.01, 95%CI: 0.77-1.10; HR=0.87, *P*＜0.01, 95%CI: 0.78- 0.97）。剔除Herbst等的研究^[5]^后，贝伐单抗联合化疗方案一线治疗晚期NSCLC的OS，与单纯使用化疗方案比较，差异具有统计学意义（RR=0.90, *P*＜0.01, 95%CI: 0.81-0.98）。Johnson等研究^[3]^中对鳞癌患者进行了OS分析，而其它5项研究^[4-8]^在设计试验时未纳入鳞癌患者，因此对这5项研究^[4-8]^高剂量贝伐单抗（15 mg/kg）联合化疗方案进行合并分析，差异具有统计学意义（HR=0.87, *P*＜0.01, 95%CI: 0.77-0.97）。

**4:**
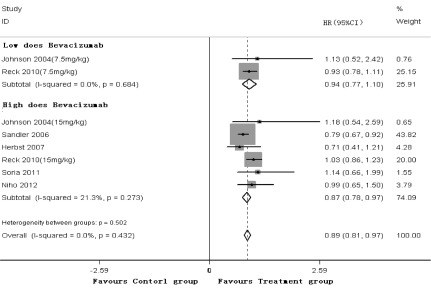
OS的*meta*分析 *Meta*-analysis of OS

#### 2.5.4 治疗相关死亡率

6项研究^[3-8]^均报道了治疗相关死亡率，*meta*分析结果（图 5）显示，低剂量贝伐单抗（7.5 mg/kg）联合化疗方案与单纯化疗方案比较，差异无统计学意义（RR=1.20, *P*=0.598, 95%CI: 0.60-2.41）；但是高剂量贝伐单抗（15 mg/kg）却明显增加治疗相关死亡率，差异具有统计学意义（RR=1.88, *P*=0.01, 95%CI: 1.16-3.05）。

**5:**
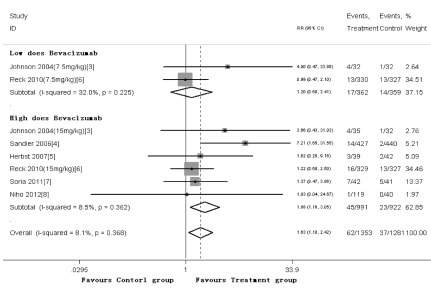
治疗相关死亡率的*meta*分析 *Meta*-analysis of treatment related mortality

#### 2.5.5 毒性反应

与单纯化疗方案比较，贝伐单抗联合化疗方案增加了患者白细胞减少、消化道反应及出血事件的发生率，而高剂量贝伐单抗（15 mg/kg）联合化疗方案还增加了高血压及蛋白尿的发生率，见表 3。

**3:** 3/4级毒性反应的*meta*分析 *Meta*-analysis of grade 3/4 toxicities

Toxicities	Quantity (*n*)	Observation	Control	Heterogeneity analysis		Effects model	RR (95%CI)	*Z*	*P*
				*I*^2^(%)	*P*				
Low-does bevacizumab (7.5 mg/kg)									
Leukopenia	2^[3, 6]^	142/362	111/359	0	0.771	Fixed	1.27 (1.04-1.55)	2.32	0.020
GI reactions	2^[3, 6]^	29/362	14/359	0	0.789	Fixed	2.06 (1.11-3.82)	2.28	0.023
Hypertension	2^[3, 6]^	21/362	6/359	54.3	0.135	Random	1.88 (0.19-18.71)	0.54	0.590
Bleeding events	2^[3, 6]^	19/362	6/359	3.3	0.309	Fixed	2.75 (1.05-7.21)	2.06	0.039
High-does bevacizumab (15 mg/kg)									
Leukopenia	6^[3-8]^	326/990	219/876	48.8	0.196	Fixed	1.33 (1.18-1.49)	4.10	0.020
Anemia	5^[4-8]^	95/1, 286	94/1, 235	46.6	0.096	Fixed	0.94 (0.72-1.23)	0.46	0.643
GI reactions	4^[3, 5, 6, 8]^	41/522	17/459	0	0.889	Fixed	2.26 (1.31-3.89)	2.94	0.003
Proteinuria	3^[4, 6, 8]^	17/875	0/825	0.8	0.365	Fixed	11.5 (2.38-55.73)	3.03	0.002
Hypertension	6^[3-8]^	73/948	9/899	0	0.741	Fixed	6.94 (3.59-13.43)	5.79	< 0.001
Bleeding events	5^[4-8]^	38/948	10/899	0	0.743	Fixed	3.27 (1.67-6.41)	3.45	0.001

## 3 讨论

血管内皮生长因子（vascular endothelial growth factor, VEGF）是一类具有高度亲和力的糖蛋白，可与血管内皮细胞发生特异性结合，其血管通透性可使肿瘤细胞进入血液循环及淋巴系统。研究^[9-12]^显示VEGF在肿瘤的发展过程中全程表达，当与内皮细胞表面受体结合后，可调控细胞的生存、增殖和迁移，VEGF信号转导途径在肿瘤信号转导中发挥着重要作用。贝伐单抗是重组人源化IgG1抗体，由人源化的IgG1片段和源自鼠类单克隆抗体与抗原结合的互补决定区域组成，可识别VEGFR并与其结合，从而抑制VEGF的生物活性，它主要的抗肿瘤作用机制为：能使现有的肿瘤血管退化，切断肿瘤细胞生长需氧及其他营养物质供应；抑制肿瘤新生血管生成，阻断肿瘤细胞的生长和转移；驱使肿瘤血管正常化，降低肿瘤组织间渗透压，改善化疗药物向肿瘤组织内的传送，提高化疗效果^[13-15]^。

晚期NSCLC患者以化疗为主要治疗手段，标准的化疗方案是4周期-6周期铂类联合第三代细胞毒药物，其有效率为20%-30%，中位生存期为8个月-10个月，疾病进展时间为3个月-5个月。ECOG 1594研究^[16]^结果显示，含铂类药物的两药联合方案治疗晚期NSCLC的生存期：紫杉醇+顺铂为7.8个月，吉西他滨+顺铂为8.1个月，多西他赛+顺铂为7.4个月，紫杉醇+卡铂为8.1个月，各方案之间无统计学差异，这些方案在一定程度上可以延长晚期NSCLC患者生存期，但疗效已达到平台期。因此，想要提高晚期NSCLC患者的有效率及生存期，需要尝试与现有治疗机制不同的新药和新的化疗方案。从2004年至今已有多项临床试验涉及贝伐单抗联合化疗治疗晚期NSCLC，显示其在延长患者PFS及OS方面较单纯采用化疗更具有优势，尤其是针对非鳞NSCLC患者。但是贝伐单抗应用于NSCLC的治疗中仍存在一定争议，尤其是ECOG4599^[4]^与AVAiL^[6]^这两项著名的多中心随机研究，虽然二者的有效率和PFS的最终结果较为相似，但二者的最终OS结果相悖。ECOG4599研究^[4]^结果显示，贝伐单抗联合卡铂和紫杉醇方案较单纯化疗延长了NSCLC患者的OS（12.3个月 *vs* 10.3个月，*P*=0.003），成为晚期NSCLC治疗史上第一个使OS提高到1年以上的方案，被视为里程碑式的突破。基于此项试验，美国NCCN指南推荐化疗联合贝伐单抗方案可用于无出血史的晚期非鳞NSCLC的治疗。AVAiL研究^[6]^中贝伐单抗（7.5 mg/kg和15 mg/kg）联合卡铂和吉西他滨治疗晚期NSCLC，与单纯化疗方案比较，OS无统计学差异（13.6个月、13.4个月 *vs* 13.1个月，*P*＞0.05），并未显示出贝伐单抗联合化疗的优势，主要的原因考虑为OS更容易受到一线治疗后的后续治疗措施的影响，即可认为ECOG4599研究^[4]^中的晚期NSCLC患者接受了更为有效的后期治疗，此外ECOG4599研究^[4]^中涉及的紫杉醇能抑制血管内皮细胞的增生，不除外与贝伐单抗具有协同作用的可能性。合并分析发现无论低剂量（7.5 mg/kg）还是高剂量（15 mg/kg）贝伐单抗联合化疗方案均可提高晚期NSCLC患者的有效率、PFS和OS。由于本文纳入的6项研究^[3-8]^均未详细报道中位PFS和OS的标准差的原始数据，因此我们合并分析了上述两项长期生存指标的效应量和95%CI，得出贝伐单抗联合化疗方案治疗晚期NSCLC可降低31%的疾病进展风险和17%的死亡风险，能使非鳞NSCLC患者临床中获益。

贝伐单抗的毒性反应也是另一被关注的焦点，尤其是治疗相关的致死性事件和出血事件。一项发表于JAMA杂志的*meta*分析^[17]^结果显示，相对于单纯化疗，贝伐单抗联合化疗的方案治疗晚期实体瘤，增加了治疗相关的死亡率。此外多中心、开放性、非随机对照的大型国际临床试验（SAiL研究）^[18, 19]^旨在获得更广泛的贝伐单抗联合化疗方案治疗晚期NSCLC的安全性和疗效数据，结果显示≥3度特征性不良事件少见，主要为血栓栓塞172例（8%），高血压125例（6%），出血80例（4%），蛋白尿67例（3%），肺出血15例（1%）。出血事件中2%暂停贝伐单抗，8%永久停用，高血压事件中7%暂停贝伐单抗，4%永久停用。本*meta*分析显示，贝伐单抗联合化疗方案治疗晚期NSCLC在一定程度上可增加毒性反应，尤其是高剂量贝伐单抗（15 mg/kg），可增加治疗相关死亡、高血压、蛋白尿、出血等不良事件的发生风险，且由于其独特的作用机制，贝伐单抗的不良反应与一般化疗药物具有不同之处，在使用贝伐单抗的过程中应严密监控患者不良反应，尤其是具有出血、胃肠道穿孔和中性粒细胞减少倾向的患者^[20, 21]^。正确选择适应症人群，治疗过程中及时发现并处理相关毒性反应，贝伐单抗联合化疗治疗晚期NSCLC可被视为有效方案。

虽然纳入的6项研究^[3-8]^均为随机对照试验，且不乏ECOG4599^[4]^及AVAiL^[6]^这样经典的大型临床研究，但是合理采用分配隐藏及盲法的研究较少，按照Cochrane协作网偏倚风险评价标准，仅AVAiL研究^[6]^一项符合“A”级标准，因此本*meta*分析存在一定的选择性偏倚。但鉴于目前肿瘤临床试验对盲法的要求较低，有效率和中位生存时间是比较客观的衡量指标，故本*meta*分析结果尚稳定可靠，可为临床制定治疗方案提供循证医学依据。同时，我们也希望今后涌现出更多大样本含量、多中心、随机对照的临床试验，能详细报道患者生存期和生活质量，进行更长时间的随访观察并详细描述终点指标，以进一步验证贝伐单抗联合化疗方案治疗晚期NSCLC的有效性和安全性。
